# Integrating miRNA and mRNA Profiling to Assess the Potential miRNA–mRNA Modules Linked With Testicular Immune Homeostasis in Sheep

**DOI:** 10.3389/fvets.2021.647153

**Published:** 2021-05-25

**Authors:** Taotao Li, Xia Wang, Ruirui Luo, Xuejiao An, Yong Zhang, Xingxu Zhao, Youji Ma

**Affiliations:** ^1^College of Animal Science and Technology, Gansu Agricultural University, Lanzhou, China; ^2^Sheep Breeding Biotechnology Engineering Laboratory of Gansu Province, Minqin, China; ^3^College of Veterinary Medicine, Gansu Agricultural University, Lanzhou, China

**Keywords:** Tibetan sheep, testis, immune privilege, blood-testis barrier, RNA-seq, miRNA

## Abstract

Beyond its well-known role in spermatogenesis and androgen production, mammalian testes are increasingly recognized as an immune-privileged organ for protecting autoantigenic germ cells, especially meiotic and postmeiotic germ cells, from systemic immune responses. Despite its importance, the molecular mechanisms underlying this regulation in mammals, including sheep, are far from known. In this study, we searched for the genes associated with testicular immune privilege and assessed their possible modulating mechanisms by analyzing systematic profiling of mRNAs and miRNAs on testicular tissues derived from prepubertal and postpubertal Tibetan sheep acquired by RNA sequencing. We identified 1,118 differentially expressed (DE) mRNAs associated with immunity (245 increased mRNAs and 873 decreased mRNAs) and 715 DE miRNAs (561 increased miRNAs and 154 decreased miRNAs) in postpubertal testes compared with prepuberty. qPCR validations for 20 DE mRNAs and 16 miRNAs showed that the RNA-seq results are reliable. By using Western blot, the postpubertal testes exhibited decreased protein abundance of CD19 and TGFBR2 (two proteins encoded by DE mRNAs) when compared with prepuberty, consistent with mRNA levels. The subsequent immunofluorescent staining showed that the positive signals for the CD19 protein were observed mainly in Sertoli cells and the basement membrane of pre- and postpubertal testes, as well as the prepubertal testicular vascular endothelium. The TGFBR2 protein was found mostly in interstitial cells and germ cells of pre- and postpubertal testes. Functional enrichment analysis indicated that DE mRNAs were mainly enriched in biological processes or pathways strongly associated with the blood–testis barrier (BTB) function. Many decreased mRNAs with low expression abundance were significantly enriched in pathways related to immune response. Also, multiple key miRNA-target negative correlation regulatory networks were subsequently established. Furthermore, we verified the target associations between either oar-miR-29b or oar-miR-1185-3p and ITGB1 by dual-luciferase reporter assay. Finally, a putative schematic model of the miRNA-mRNA-pathway network mediated by immune homeostasis-related genes was proposed to show their potential regulatory roles in sheep testicular privilege. Taken together, we conclude that many immune-related genes identified in this study are negatively regulated by potential miRNAs to participate in the homeostatic regulation of testicular immune privilege of sheep by sustaining BTB function and inhibiting immune responses under normal physiological conditions. This work offers the first global view of the expression profiles of miRNAs/mRNAs involved in sheep testicular immune privilege and how the genes potentially contribute to immune-homeostatic maintenance.

## Introduction

Tibetan sheep (*Ovis aries*) provide food and livelihood to the inhabitants of Tibet (1) and play an important role in the functions of the Tibetan ecosystem (2). However, this breed of sheep is slow to mature. Thus, insight into the gonad development (including homeostasis of the internal environment) of Tibetan sheep is of great practical importance to the reproductive biology of sheep and other domestic animals.

The testes are an extremely important reproductive organ that determines male fertility. They contain spermatogenic cells that can maintain the production of male gametes. Immunologically, the testes are also perceived as an immune-privileged organ (3). Protection of the immunogenic spermatogenic cells (especially meiotic and postmeiotic cells) from the host immune response is fundamental to guarantee continuous spermatogenesis and male fertility. This process has been increasingly reported to rely on this privilege (4, 5). Except germ cells (GCs), almost all testicular cell types including Sertoli cells (SCs) (6), myoid cells (7), Leydig cells, and immune cell populations, such as macrophages, lymphocytes (mainly T cells), dendritic cells (DCs), and mast cells (3, 8), are regarded as possessing immunoregulatory properties, collectively participating in the formation of testicular immune privilege. On the one hand, testicular immune privilege prevents immunogenic GCs from autoimmune responses, while on the other hand, it protects GCs against inflammatory responses and microbial infections (3). Previous studies have documented that testicular immune privilege is tightly regulated by numerous genes, such as tight junction protein catenins (CTNNs), occludins (OCLNs), and claudins (CLDNs) (9–11), and transforming growth factor β (TGFB) family members (3, 12). Nevertheless, the genes that control testicular immune privilege in sheep and their expression modulation are poorly known.

MicroRNAs (miRNAs), a class of endogenous short non-coding small RNAs (sRNAs), can modulate the expression patterns of genes at the transcriptional and posttranscriptional levels. miRNAs participate in almost all essential biological and physiological processes, including reproductive process (13) and development of the immune system (14). Additionally, current research on miRNAs involved in reproductive immunity has mainly focused on the female reproductive tract (15, 16) and is highly limited in terms of the testes (17), especially sheep testes.

RNA sequencing (mRNA-seq and sRNA-seq) has emerged as a powerful tool to identify and characterize the genes and miRNAs expressed in mammalian testes (18, 19). RNA sequencing is of great significance for further filtering the immune-related mRNAs–miRNAs in testes and understanding the complex processes that modulate the maintenance of the testes' immune privilege. Based on this background, we hypothesized that genes participating in the regulation of immune privilege maintenance in ram testes could be identified on the background of gene expression changes due to other cellular processes involved in sexual maturation of testis tissue. By filtering genes with differential expression between prepubertal and sexually mature ram testes according to their proposed involvement in immune-related processes, we are attempting to focus on the biological mechanisms involved in immune privilege development. We further hypothesized that specific miRNAs with potential involvement in regulating these genes could be identified in parallel. This study was therefore conducted by RNA-seq analysis combined with molecular biological experiments to screen and mine the immune-related genes that show differential abundance in prepubertal and postpubertal ram testes. Besides, we explore if the genes that participate in testicular immune privilege are regulated by miRNAs, thus, contributing to understanding the complex processes that modulate the maintenance of testis immune privilege.

## Materials and Methods

### Experimental Animals and Sampling

A total of 16 healthy male Tibetan sheep derived from the same ram was selected based on their birth records and divided into two age groups: sexual immaturity (3-month old; *n* = 8) and sexual maturity (1-year old; *n* = 8). All animals were purchased from the Ganjia Tibetan Sheep Breeding Cooperative (Xiahe, Gansu, China). Following sacrifice, the right testicular tissues were obtained from all the sheep of the two age groups (testes from 3-month-old sheep, T3M; testes from 1-year-old sheep, T1Y) and then divided in the following two parts: one part was stored at −80°C used for RNA and protein extraction, and the other was fixed in 4% paraformaldehyde for at least 24 h, embedded in paraffin, and sectioned in 5-μm paraffin sections.

### RNA Extraction

Total RNA of each testis sample was extracted using Trizol reagent (Invitrogen, Carlsbad, CA, USA) according to the kit's operation manual. RNA quality was evaluated first by 1.0% agarose electrophoresis, then determined on an Agilent 2100 Bioanalyzer (Agilent Technologies, Palo Alto, CA, USA). All samples had an RNA integrity number (RIN) >7.5. Of the RNA samples from eight rams for each age group, four were randomly selected and used to construct cDNA libraries for mRNA and sRNA sequencing, and all eight RNA samples from each group were used for qPCR validation.

### Library Preparation for mRNA-seq and Data Processing

The 470- to 500-bp size ligation products were enriched to generate a strand-specific cDNA library for mRNA-seq. Construction of the mRNA libraries was performed as described earlier (20). A total of eight prepared cDNA libraries from two age groups were sequenced on Illumina HiSeq™ 4000 by Gene Denovo Biotechnology Co., Ltd. (Guangzhou, China). The high-quality clean reads were screened from the raw reads by trimming and filtering reads with adaptors, more than 10% of unknown nucleotides (N), and low-quality reads with more than 50% of low-quality (q-value ≤20) bases. The clean reads of each sample were first filtered for ribosomal RNAs and then mapped to the *Ovis aries* reference genome (Oar_v4.0) by TopHat2 (version 2.1.1) using default parameters (21).

### Library Preparation for sRNA Sequencing and Data Processing

The 140- to 160-bp size ligation products were enriched to generate a cDNA library for sRNA sequencing. A total of eight cDNA libraries from two age groups were sequenced on the Illumina HiSeq™ 2500 platform by Gene Denovo Biotechnology Co., Ltd. (Guangzhou, China). The clean tags were obtained from the raw reads by filtering out the low-quality reads with more than one low quality (q-value ≤20) base or containing unknown nucleotides (N), and reads without 3′ adapters, reads containing 5′ adapters, 3′ and 5′ adapters but no small RNA fragment between them, polyA in small RNA fragment, and reads with lengths shorter than 18 nt (not including adapters). All of the clean tags were aligned with small RNAs in the GenBank database (http://blast.ncbi.nlm.nih.gov) and Rfam database (http://sanger.ac.uk/software/Rfam) to identify and discard the cellular structural RNAs (rRNA, snRNA, snoRNA, and tRNA).

### Identification of Known and Novel miRNAs

All of the clean tags were mapped to the miRBase 21.0 database (http://www.mirbase.org/) to identify known ovine miRNAs. The remaining clean tags that were not mapped to the sheep miRBase were then mapped to the other animal species included in the miRBase database to identify the known miRNAs. For all of the other unannotated tags aligned with the reference genome using Bowtie (v1.1.2), the novel miRNAs were predicted by software Mireap_v0.2 and identified according to their genome positions and hairpin structures. The default parameters were used in all software.

### Screening of Differentially Expressed mRNAs and miRNAs

mRNA abundances were quantified via the software RSEM (22), and their expression levels in each sample were normalized by using the FPKM method (23). The miRNA expression level from each sample was calculated and normalized to TPM (TPM = actual miRNA count/total count of clean reads × 10^6^). Differentially expressed (DE) mRNAs and miRNA analyses between the two age groups were performed using the edgeR package (http://www.bioconductor.org/packages/release/bioc/html/edgeR.html). We identified DE mRNAs with an absolute fold change >2 and FDR < 0.05, and DE miRNAs with an absolute fold change >2 and *p*-value < 0.05.

### Functional Enrichment of Differentially Expressed mRNAs and Screening of Immune-Related Differentially Expressed Genes

Gene Ontology (GO) annotation and Kyoto Encyclopedia of Genes and Genomes (KEGG) enrichment analysis for all DE mRNAs were performed with the GO database (http://www.geneontology.org/) and KEGG database (http://www.genome.jp/kegg/pathway.html), respectively. GO terms and pathways with a q-value < 0.05 were considered significantly enriched by DE mRNAs. To explore the potential function of some genes implicated in the maintenance of testicular immune privilege, the immune-related DE mRNAs were filtered based on the results from the above GO and KEGG analysis, and again mapped to GO terms in the GO database and pathways in the KEGG database.

### Target Prediction of Differentially Expressed miRNAs and Integrative Analysis of Immune-Related miRNA–mRNA Pairs

The candidate target genes of DE miRNAs were predicted by using the RNAhybrid (v2.1.2) + svm_light (v6.01), Miranda (v3.3a), and TargetScan (v 7.0) software. The intersection of the results from three software packages were selected as predicted miRNA target genes. Expression correlation between miRNA and its predicted target was assessed by Pearson correlation coefficient (PCC). Subsequently, the negatively coexpressed immune-related miRNA–mRNA pairs with PCC < −0.7 and *p*-value < 0.05 were screened to construct miRNA–mRNA networks.

### Validation of Differentially Expressed mRNAs and miRNAs Using Quantitative Real-Time PCR

Total RNA was extracted from all 16 testis samples of two age groups using Trizol reagent (TransGen, Beijing, China). The first-strand cDNA for mRNAs was synthesized from 500 ng of each total RNA sample using a TransScript II All-in-One First-Strand cDNA Synthesis SuperMix (TransGen Biotech, Beijing, China) following the manufacturer′s recommendations. cDNA synthesis for miRNAs was performed from 500 ng of each total RNA sample using a Mir-X™ miRNA FirstStrand Synthesis Kit (Takara, Shiga, Japan) according to the kit instructions. The qPCR was carried out using a TB Green™ Fast qPCR Mix (Takara, Shiga, Japan) on a LightCycler 96 Real-Time System (Roche, Switzerland). The specific primers used in qPCR were designed and synthesized by the Qingke Biological Company (Xi'an, China). Primer sequences are provided in Supplementary Table 1. Eight independent biological replicates were included in qPCR analysis. β-actin and U6 were used as internal control genes for expression normalization of mRNAs and miRNAs, respectively. The relative expression levels of mRNAs and miRNAs were calculated using the 2^−ΔΔCt^ method (24).

### Western Blot

Total protein was extracted from all 16 testis samples in two age groups, and its concentration was detected using a BCA protein assay reagent (Beyotime, Shanghai, China). Western blot assay was performed, as described in a previous report (25). β-actin was used as a protein-loading control. Briefly, a total of 20 μg of protein from each sample was separated by 12% SDS-PAGE gradient gels and electrotransferred onto PVDF membranes. Membranes were incubated with rabbit polyclonal anti-CD19 antibody (Bioss, Beijing, China; 1:500 dilution), rabbit polyclonal anti-TGF beta receptor II antibody (Bioss, Beijing, China; 1:500 dilution), or rabbit polyclonal anti-beta-actin antibody (Bioss, Beijing, China; 1:1,500 dilution) and the secondary antibody goat anti-rabbit IgG conjugated with HRP (Bioss, Beijing, China; 1:5,000 dilution). Protein bands were visualized using an ECL kit (NCM Biotech, Suzhou, China) and quantified using AlphaEaseFC software (Protein Simple, Santa Clara, CA, USA).

### Immunofluorescence

Paraffin sections were deparaffinized, hydrated, and subjected to antigen retrieval. An immunofluorescence assay was carried out as described previously (25). In brief, sections were blocked with 5% bovine serum albumin (BSA) for 1 h and incubated with rabbit polyclonal anti-CD19 antibody (Bioss, Beijing, China; 1:150 dilution) or rabbit polyclonal anti-TGF beta receptor II antibody (Bioss, Beijing, China; 1:200 dilution). After being washed in PBST (PBS with 0.5% Tween-20), the sections were incubated with the secondary antibody goat anti-rabbit IgG conjugated with Cy3 or FITC. Nuclei were visualized using DAPI (Servicebio, Wuhan, China). Sections were visualized under a fluorescence microscope (Nikon, Eclipse C1, Tokyo, Japan), and images were acquired using CaseViewer software (3DHISTECH, Budapest, Hungary). For negative controls, the primary antibody was replaced with 5% BSA.

### Dual-Luciferase Reporter

The wild-type ITGB1 3′UTR fragment containing the putative miR-29b or miR-1185-3p binding site and their corresponding mutant-type fragments were designed and synthesized (GENEWIZ, Suzhou, China), and then cloned into the pmirGLO plasmid (Promega, Madison, USA) between the XhoI and SalI multicloning sites, named as ITGB1-29-3′UTR WT, ITGB1-1185-3′UTR WT, ITGB1-29-3′UTR MUT, and ITGB1-1185-3′UTR MUT, respectively. The miR-29b mimic (5′-UAGCACCAUUUGAAAUCAGUGU-3′, 5′-ACUGAUUUCAAAUGGUGCUAUU-3′), miR-1185-3p mimic (5′-AUAUACAGAGGGAGACUCUUAU-3′, 5′-AAGAGUCUCCCUCUGUAUAUUU-3′), and mimic negative control (5′-UUCUCCGAACGUGUCACGUTT-3′, 5′-ACGUGACACGUUCGGAGAATT-3′) were bought from GenePharma (Shanghai, China). The reporter plasmids were cotransfected with either the corresponding mimic or mimic NC into HEK293T cells (Beina Biology, Beijing, China) using the Lipofectamine 2000 vehicle (Invitrogen, Carlsbad, USA) as per the vendor's recommendations. Both firefly luciferase and Renilla luciferase activities were monitored at 48 h following transfection, using a dual-luciferase reporter assay system (Promega, USA). Data are shown as relative luciferase activity generated by dividing the firefly luciferase values with those of the Renilla luciferase.

### Statistical Analysis

At least three independent repeats were undertaken for each of the experiments. Comparisons between two groups were made using independent-samples *t*-tests or one-way ANOVAs in SPSS 21.0 (SPSS, Inc., Chicago, IL, USA). The results were expressed as mean ± SD. Significant differences between the two groups were considered in terms of the associated *p-*value relative to *p* < 0.05 and *p* < 0.01.

## Results

### Identification and Functional Classification of Differentially Expressed mRNAs

**Figure 1:**
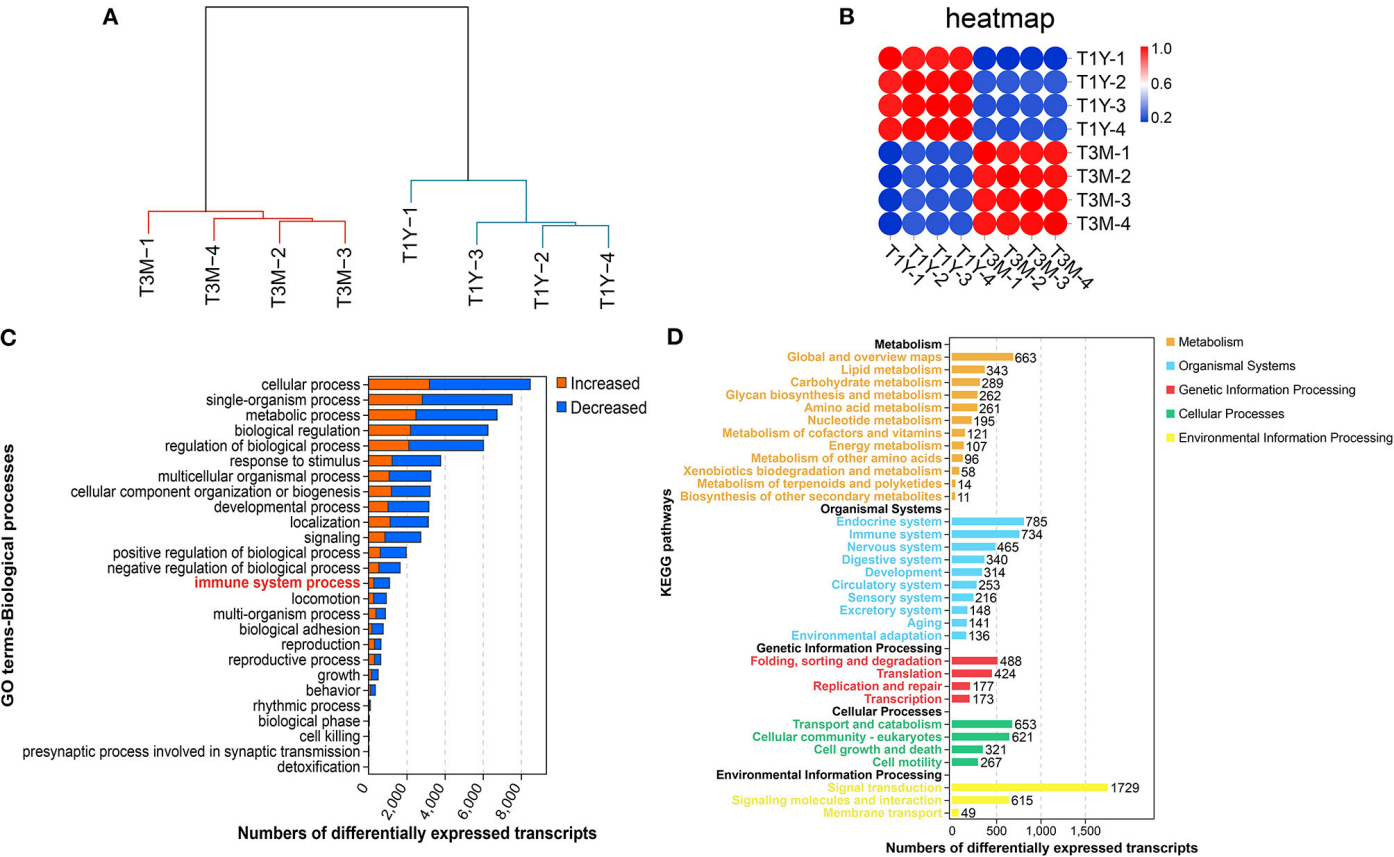
Differential gene expression analysis between T3M and T1Y. **(A)** Hierarchical clustering dendrograms of the biological duplicates. **(B)** Sample correlation heatmap. **(C)** Biological processes mediated by DE mRNAs based on Gene Ontology (GO) annotation results. **(D)** Kyoto Encyclopedia of Genes and Genomes (KEGG) enrichment analysis for DE mRNAs. T3M, testes from 3-month-old sheep; T1Y, testes from 1-year-old sheep; DE, differentially expressed.

We carried out mRNA-seq analysis and obtained an average of 85, 301, 018 (99.04% of raw reads) and 78, 331, 884 high-quality clean reads (98.27% of raw reads) from T3M and T1Y, respectively, after removal of rRNA and low-quality reads. Of the high-quality clean reads, on average, 77.17% of the reads were uniquely mapped to the ovine reference genome. The detailed statistics for each library are listed in Supplementary Table 2. The hierarchical clustering results showed that the samples from T3M and T1Y groups separated into two distinct clusters (Figure 1A). The sample correlation heat map from the mRNA expression profiles indicated that four replicate samples from each group had good repeatability (Figure 1B). Here, we identified 26,084 DE mRNAs (17,472 genes), of which 10,247 were more abundant, while 15,837 were less abundant in the T1Y group (|fold change| > 2, FDR < 0.05). GO and KEGG enrichment analysis showed that most DE genes were mainly involved in the biological processes and pathways related to cell growth, reproduction, immunity, and metabolism (Figures 1C,D).

### Screening of Differentially Expressed mRNAs Related to Immunity

**Figure 2:**
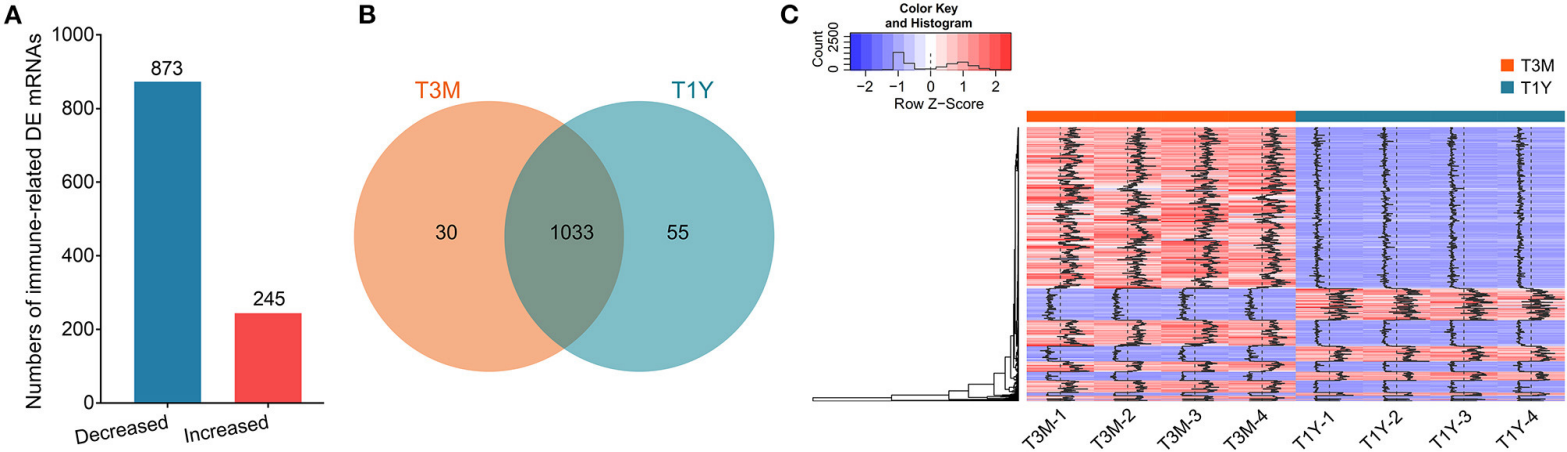
Filtering of DE mRNAs related to immunity. **(A)** The histogram shows the numbers of the immune-related DE mRNAs. Red and blue represent increased and decreased mRNA abundance in T1Y, respectively. **(B)** Venn diagram of the immune-related DE mRNAs in two age groups. **(C)** The clustering heatmap for the immune-related DE mRNAs. T3M, testes from 3-month-old sheep; T1Y, testes from 1-year-old sheep; DE, differentially expressed.

To explore the potential functions of genes involved in maintaining testis' immune privilege during development, we further screened the immune-related DE mRNAs. Herein, we identified 1,118 DE mRNAs that were associated with immunity. Of these, 245 mRNAs exhibited increased abundance in the T1Y group relative to the T3M group, with the remaining 873 mRNAs exhibiting decreased abundance (Figures 2A,B). Clustering heatmap analysis for these mRNAs showed excellent repeatability and gene expression profiles for the different age groups (Figure 2C). For a detailed list of all these mRNAs and their expression levels, see Supplementary Table 3.

### Functional Annotation of Immune-Related Genes

**Figure 3:**
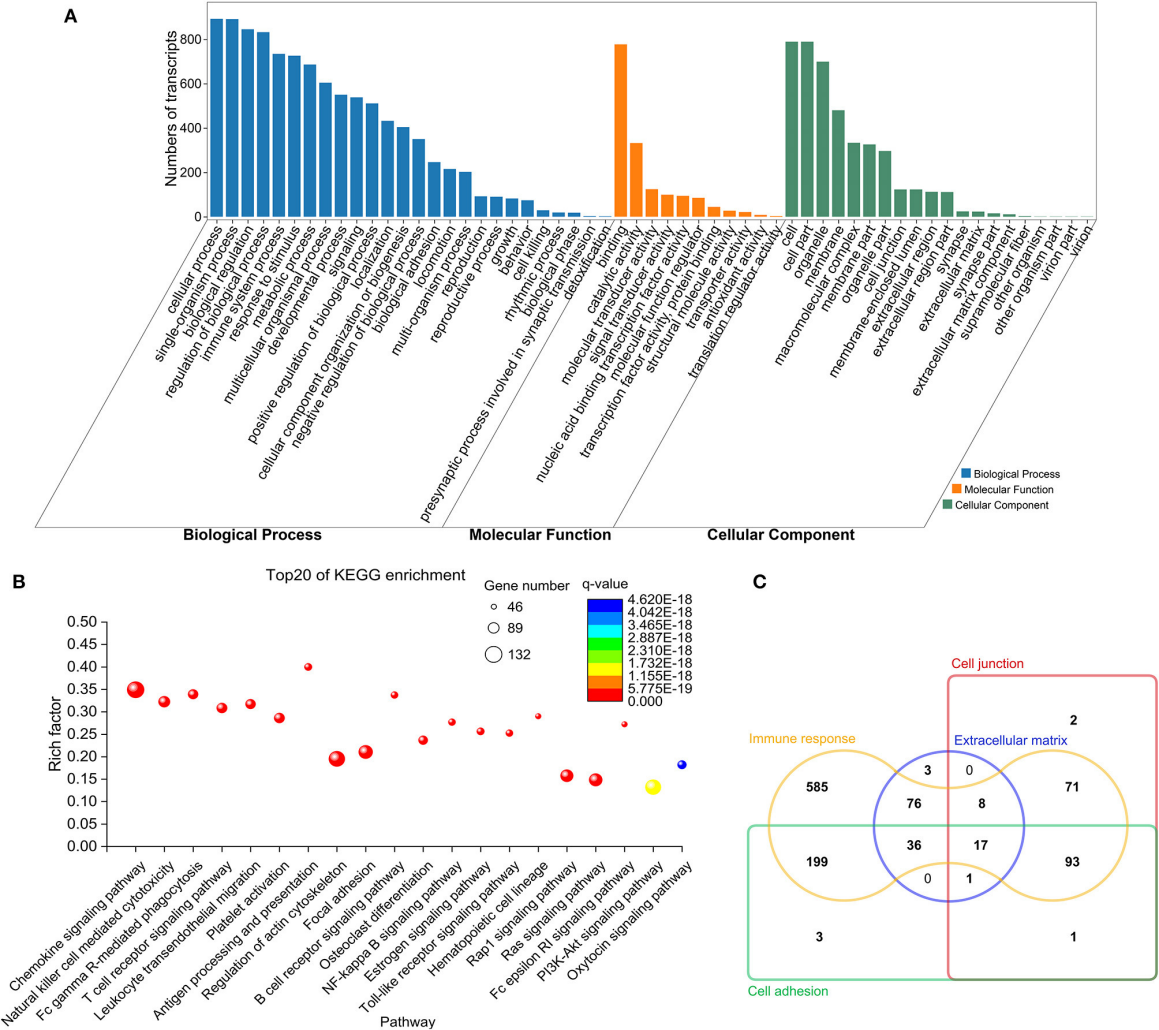
Functional annotation and enrichment analysis for immune-related DE mRNAs. **(A)** GO annotation results. **(B)** Top 20 KEGG pathways enriched by immune-related mRNAs. Y-axis denotes the enrichment factor, which was calculated by dividing the number of enriched genes (mRNAs) in the KEGG pathway by the number of annotated genes in this pathway. **(C)** Venn diagram of DE mRNAs related to extracellular matrix remodeling, cell junction, cell adhesion, and immune response.

GO analysis showed that these immune-related genes, in the category of biological process, were mainly involved in immune system processes, cell adhesion/junction, responding to stimulus and immune responses; in the category of cell component, they were mainly implicated in the cell junction, extracellular matrix (ECM), membrane region, cell part, and receptor complexes; and in the category of molecular function, they were mainly associated with protein binding, receptor binding, receptor regulator activity, and cytokine receptor activity (Figure 3A and Supplementary Table 4). KEGG enrichment results revealed that most of these genes were significantly enriched in pathways associated with the immune system, such as the chemokine signaling pathway, T- and B-cell receptor pathways, leukocyte trans endothelial migration, NF-κB signaling, and focal adhesion (Figure 3B and Supplementary Table 5). There were 17 DE mRNAs (corresponding to 14 DE genes) associated with four functions: immune response, presence at the cell junction, ECM remodeling, and cell adhesion (Figure 3C).

### Identification and Analysis of DE miRNAs

**Figure 4:**
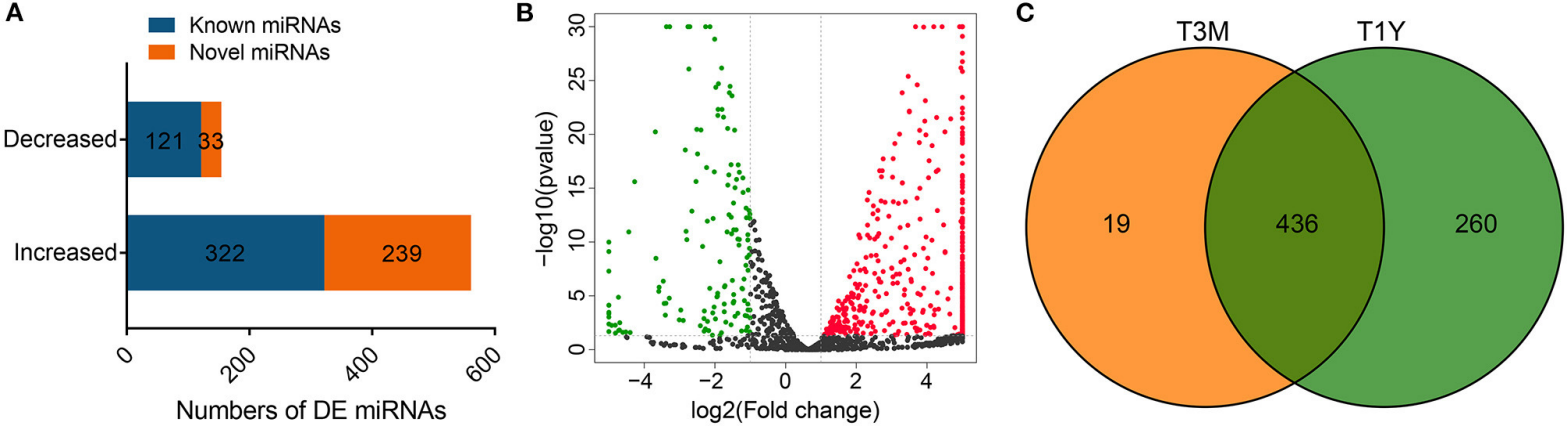
Analysis of DE miRNAs. **(A)** Numbers of DE miRNAs. **(B)** Volcano plot of DE miRNAs. Red represents increased miRNA abundance, and green represents decreased miRNA abundance in T1Y relative to T3M. **(C)** Venn diagram representation of the common and group-specific DE miRNAs. T3M, testes from 3-month-old sheep; T1Y, testes from 1-year-old sheep; DE, differentially expressed.

After removing low-quality reads and adapter sequences, an average of 12, 737, 654 (95.45% of raw reads) and 10, 898, 503 (79.13% of raw reads) clean tags were obtained from the four samples of the T3M group and four samples of the T1Y, respectively. Of the clean tags, 81.70% of the tags from the T3M and 73.25% of the tags from the T1Y were mapped to the reference sequence. The detailed statistics for each library are given in Supplementary Table 6. A total of 715 DE miRNAs, including 561 increased and 154 decreased miRNAs, were obtained in the T1Y group compared with the T3M group (Figures 4A,B), in which 443 known miRNAs and 272 novel miRNAs were identified (Figure 4A). Among these DE miRNAs, 19 miRNAs were specifically expressed in the testes from the T3M group, 260 miRNAs were specifically expressed in the testes from the T1Y group, and 436 miRNAs were expressed in both (Figure 4C). Detailed information on miRNA expression is given in Supplementary Table 7.

### Interaction Network of miRNAs and Target mRNAs Associated With Immunity

**Figure 5:**
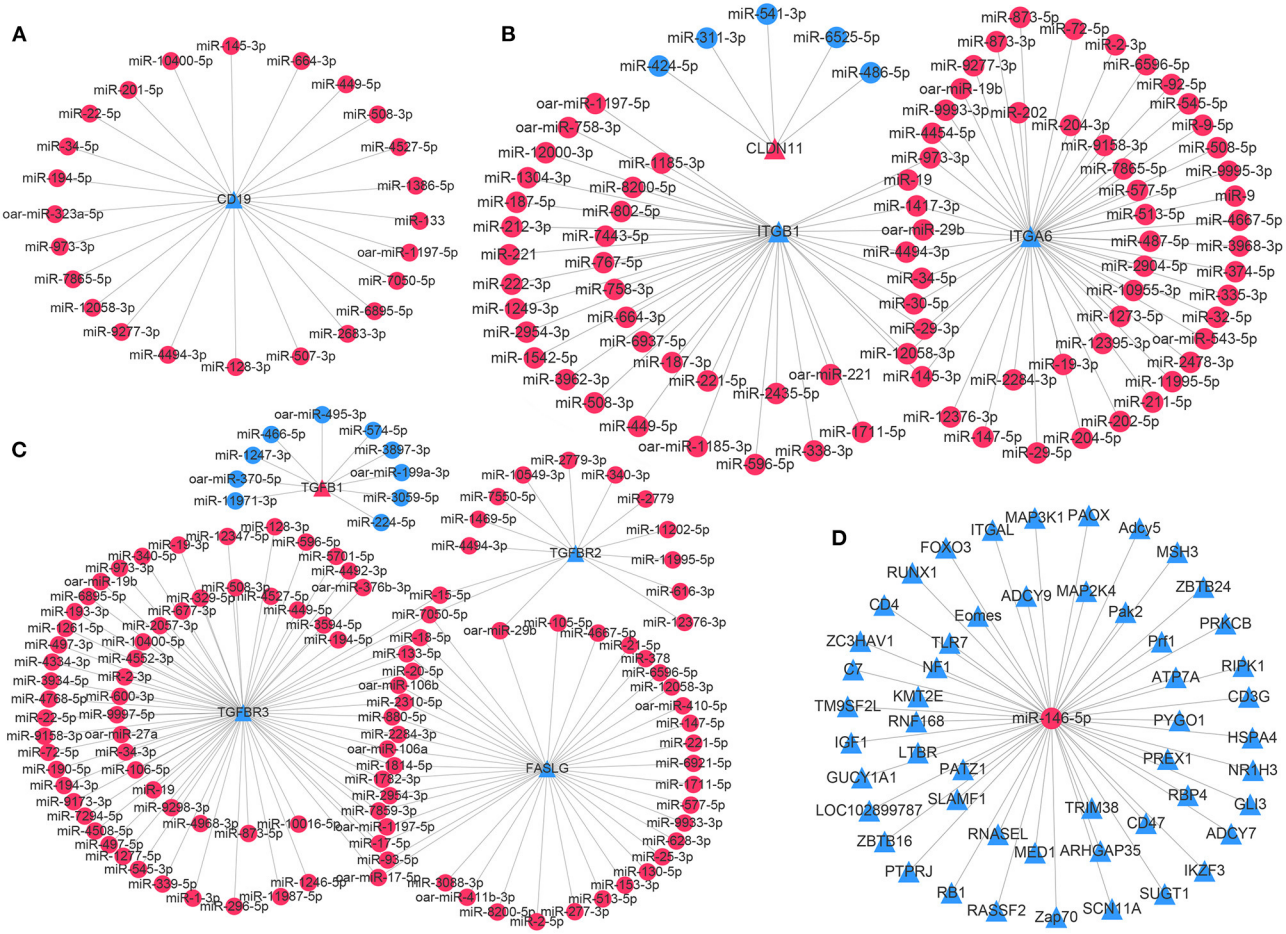
Integrated miRNA-target negative correlation regulatory networks. **(A)** Immune-related gene CD19 was potentially regulated by 24 known miRNAs. **(B)** Three genes related to the blood–testis barrier function, namely, CLDN11, ITGA6, and ITGB1, were potentially regulated by 87 known miRNAs. **(C)** Four genes that have been reported as testicular immune homeostasis-related genes, including TGFB1, TGFBR2, TGFBR3, and FASLG, were potentially regulated by 117 known miRNAs. **(D)** Fifty-one DE genes were potentially regulated by miR-146-5p, an miRNA related to immune homeostasis maintenance. The circle nodes represent miRNAs; the triangle nodes suggest target genes. The differentially expressed miRNAs/target genes are highlighted in red and blue, describing higher and lower expression in the T1Y group compared with the T3M group, respectively. T3M, testes from 3-month-old sheep; T1Y, testes from 1-year-old sheep.

To explore potential miRNA target transcripts involved in the homeostatic regulation of testicular immune privilege, the expression profiles of DE miRNAs and immune-related DE mRNAs were combined for further correlation analysis. In total, we obtained 554 DE mRNAs, corresponding to 471 DE genes as putative targets for 714 DE miRNAs (442 known and 272 novel miRNAs) through integrated analysis, presenting a negatively correlated expression pattern (Supplementary Table 8). DE mRNAs (564) were not found to have potential target miRNAs. The resulting key potential regulatory networks of miRNA-target genes associated with testicular immune privilege were visualized with the Cytoscape software (version 3.7.1). For instance, a total of 24 known miRNAs were potentially targeted for CD19, a gene implicated in immune response (Figure 5A). Three BTB maintenance-related genes (CLDN11, ITGA6, and ITGB1) were potentially targeted by 87 known miRNAs, of which ITGA6 and ITGB1 were shared by 10 miRNAs (Figure 5B). Four genes associated with testicular immune homeostasis (TGFB1, TGFBR2, TGFBR3, and FASLG) were potentially regulated by 117 known miRNAs, in which FASLG and TGFBR3 were coregulated by 17 miRNAs (Figure 5C). miR-146-5p (an immune homeostasis maintenance-related miRNA) was identified as potentially involved in regulating the expression of 51 DE mRNAs (Figure 5D).

### qPCR Validation of DE mRNAs and DE miRNAs

**Figure 6:**
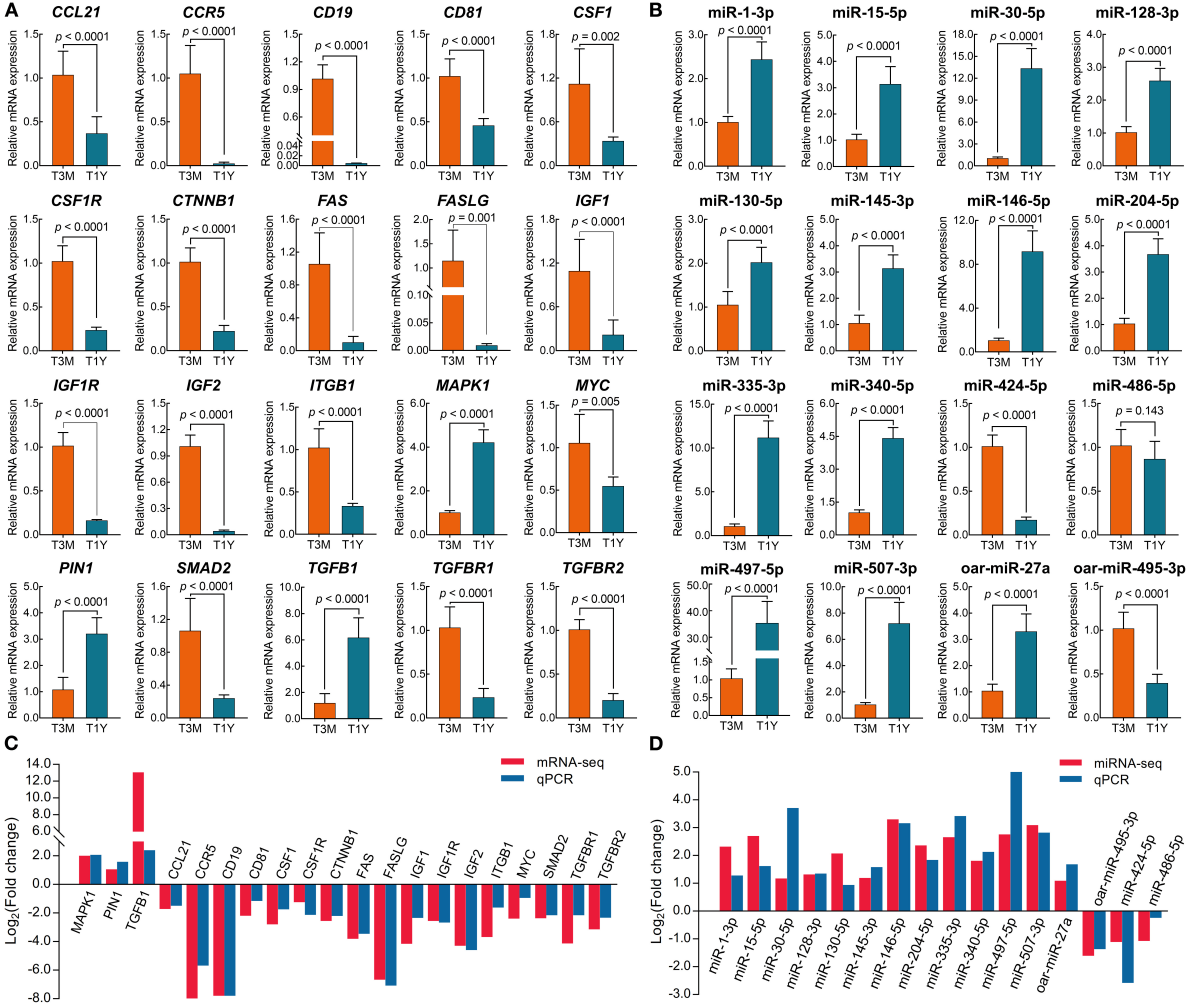
Verification of DE mRNAs and DE miRNAs by qPCR. **(A)** qPCR analysis for 20 randomly selected mRNAs. Data represent the mean ± SD. **(B)** qPCR analysis for 16 randomly selected miRNAs. Data represent the mean ± SD. **(C)** The comparison of mRNA expression in terms of Log_2_ (fold change) as assessed by mRNA sequencing and qPCR. **(D)** The comparison of mRNA expression in terms of the Log_2_ (fold change) as assessed by miRNA sequencing and qPCR. T3M, testes from 3-month-old sheep; T1Y, testes from 1-year-old sheep; DE, differentially expressed.

To verify the results of mRNA-seq and miRNA-seq, 20 DE mRNAs and 16 DE miRNAs were randomly selected for qPCR analysis. The results showed that the expression of all the selected mRNAs and miRNAs except for miR-486-5p was significantly different between the two age groups (*p* < 0.01) (Figures 6A,B). Overall, the expression trends of mRNA and miRNA upregulation or downregulation revealed by the qPCR data were consistent with those derived from RNA sequencing data (Figures 6C,D), which indicates that our transcriptome data were credible for identification of the differentially expressed mRNAs and miRNAs.

### Expression and Localization Patterns of Proteins Encoded by Immune-Related Genes

**Figure 7:**
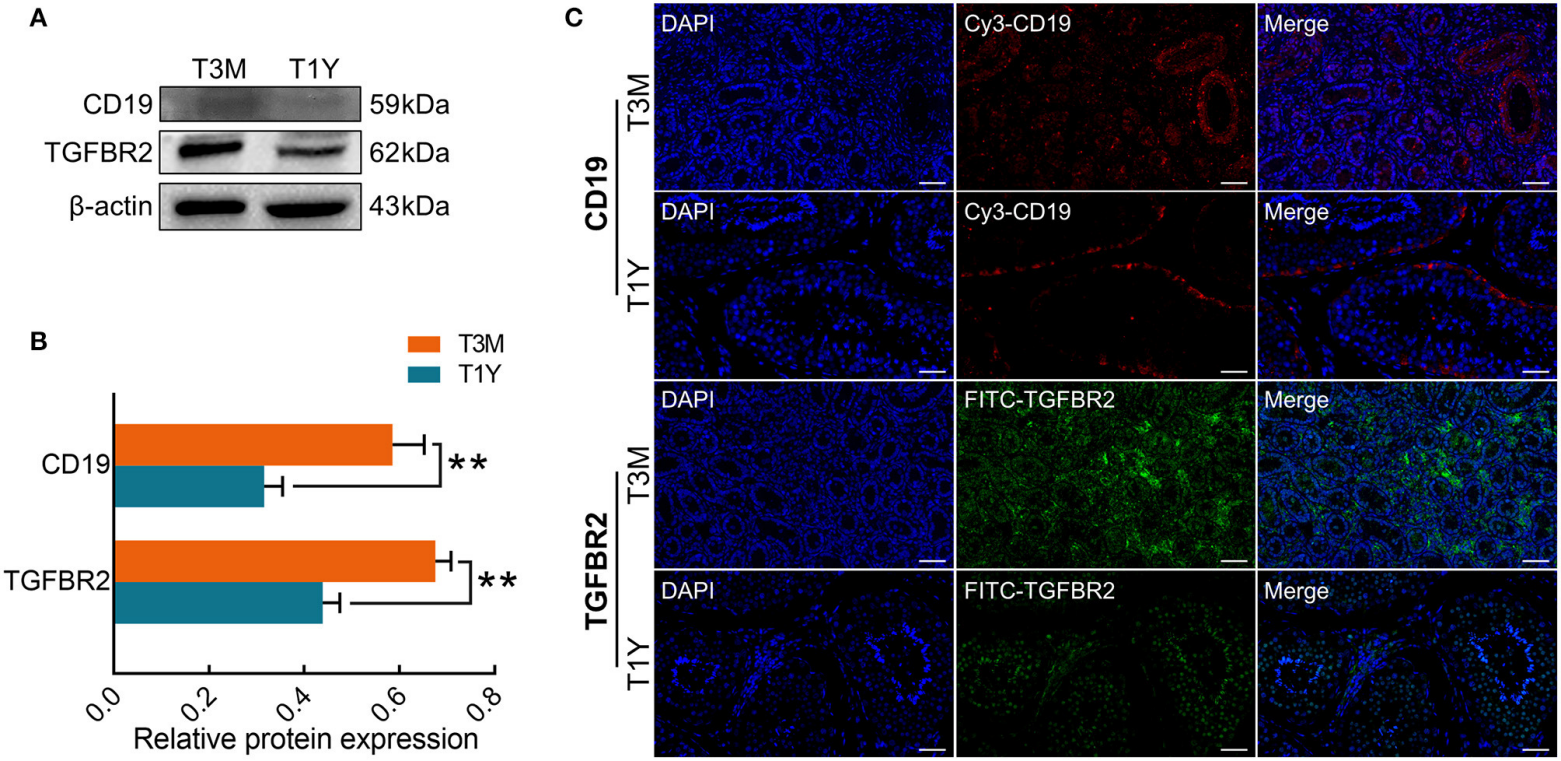
Expression and localization patterns of CD19 and TGFBR2 proteins. **(A)** Western blot analysis. **(B)** Relative protein expression. Data represent the mean ± SD. **(C)** Immunofluorescence staining. Blue, DAPI; red/CY3, CD19; green/FITC, TGFBR2. Scale bar, 50 μm. T3M, testes from 3-month-old sheep; T1Y, testes from 1-year-old sheep; DE, differentially expressed. **P < 0.01.

To determine gene expression status at the protein level, Western blot analysis was performed for two immune-related genes (CD19 and TGFBR2). The results showed that CD19 and TGFBR2 proteins were downregulated in the T1Y group compared with the T3M group (Figures 7A,B), which exhibited a similar trend to those at the mRNA level. To explore the potential roles of CD19 and TGFBR2 in developmental sheep testes, and their localization patterns in sexual immaturity (T3M) and maturity (T1Y), testes were assayed by immunofluorescence. As shown in Figure 7C, stronger positive signals for the CD19 protein were observed in vascular endothelium from the T3M group as well as SCs and the basement membranes from the T3M and T1Y groups, and weak positive signals were observed in interstitial cells from the T3M group. For the TGFBR2 protein, strong positive signals were distributed in the interstitial cells of both T3M and T1Y, and moderate positive signals were also observed in gonocytes and spermatogonia from T3M, as well as spermatogonia, spermatocytes, and spermatids from T1Y.

### Validation of Targeting Relations Between Either miR-29b or miR-1185-3p and ITGB1

**Figure 8:**
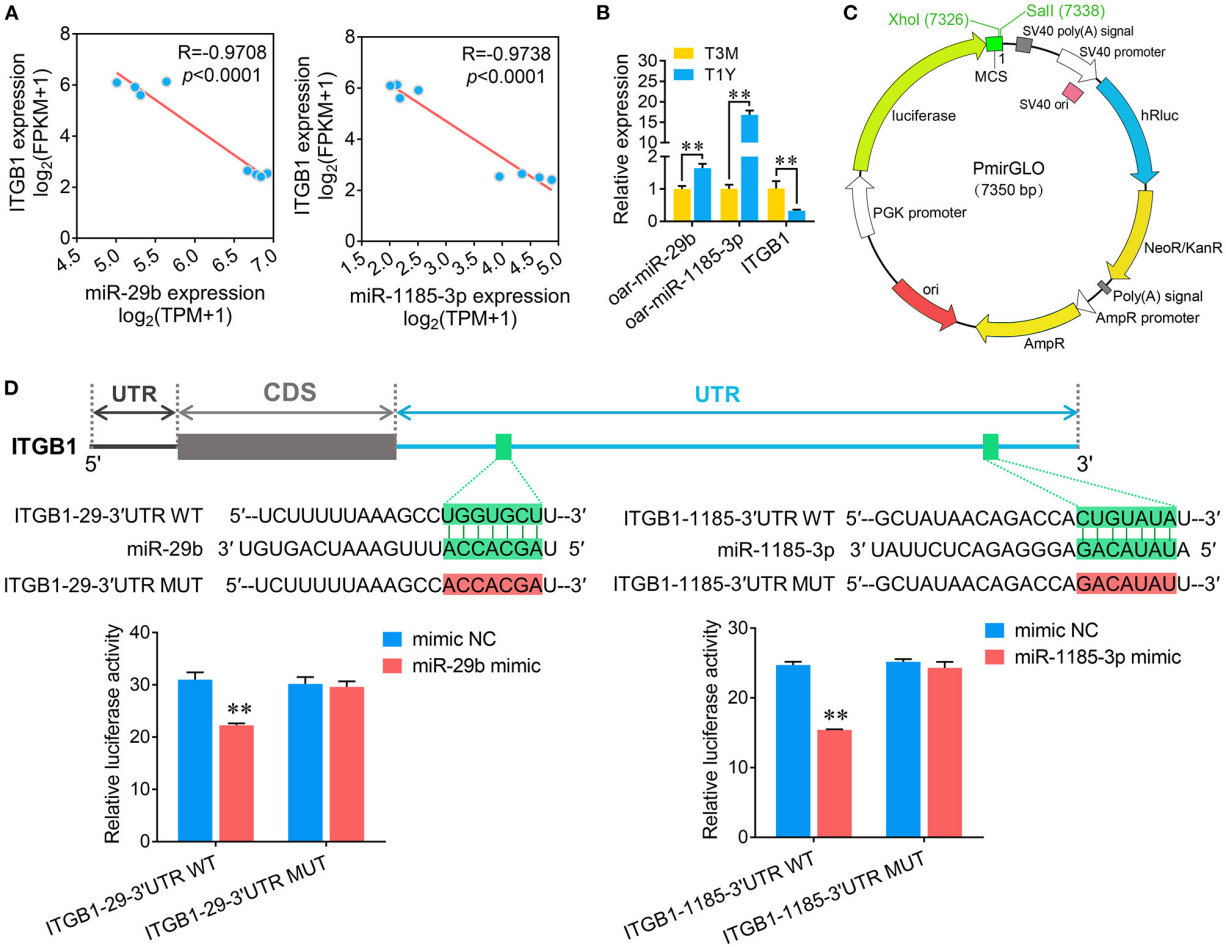
Validation of targeted associations between either miR-29b or miR-1185-3p and ITGB1. **(A)** Pearson correlation assessment between oar-miR-29b/oar-miR-1185-3p and ITGB1 expression. **(B)** Quantitative real-time PCR (qPCR) detection for oar-miR-29b, oar-miR-1185-3p, and ITGB1 expression. **(C)** Schematic presentation of the pmirGLO dual-luciferase miRNA target expression vector used for constructing ITGB1-3′UTR. **(D)** A dual-luciferase reporter assay verified the interaction between miR-29b/miR-1185-3p and ITGB1. (Upper panel) The binding sites on ITGB1 3′-UTR and the seed region of miR-29b or miR-1185-3p. (Lower panel) Relative luciferase activity was measured by a dual-luciferase reporter assay. Data represent the mean ± SD. T3M, testes from 3-month-old sheep; T1Y, testes from 1-year-old sheep. **P < 0.01.

ITGB1, a well-known cell adhesion-associated molecule that is implicated in the function of the blood–testis barrier (26), was discovered to be potentially regulated by 40 known miRNAs based on miRNA–mRNA coexpression network analysis. Among these, high abundance miR-29b and low abundance miR-1185-3p were randomly chosen to verify the targeting relationships between them and ITGB1. First, Pearson correlations revealed that ITGB1 expression exhibited a strong negative correlation with either miR-29b or miR-1185-3p (Figure 8A). Validation by qPCR confirmed a significant increase in miR-29b or miR-1185-3p and a significant decrease in ITGB1 mRNA observed in the RNA-seq analysis (Figure 8B). To validate whether ITGB1 is a genuine miR-29b and miR-1185-3p target, we employed a luciferase reporter assay by constructing the 3′UTR of ITGB1 containing the putative miR-29b or miR-1185-3p binding sites into XhoI and SalI sites of the pmirGLO dual-luciferase miRNA target expression vector (Figure 8C). As demonstrated in Figure 8D, the luciferase reporter activity for wild-type ITGB1 3′UTR (ITGB1-29-3′UTR WT and ITGB1-1185-3′UTR WT) rather than the mutant ones, was significantly reduced following transfection with either miR-29b or miR-1185-3p (Figure 8D). Together, these data initially corroborated that ITGB1 is a common target of miR-29b and miR-1185-3p.

## Discussion

To identify the potential genes involved in sheep testes' immune privilege, mRNA-seq was carried out for testis tissues derived from T3M (pre-puberty) and T1Y (post-puberty) individuals. We identified a total of 1,118 immune-related DE mRNAs corresponding to 208 genes with increased abundance and 725 genes with decreased abundance in the T1Y group compared with the T3M group. Functional bioinformatics analysis based on the GO and KEGG database showed that these genes were mainly involved in various biological processes or pathways related to immune response, tight junction, cell adhesion, and ECM organization. To understand the possible modulating mechanisms of the genes implicated in testicular immune privilege, the subsequent miRNA-seq was carried out, and miRNA–mRNA regulatory networks were constructed by merging miRNA–mRNA pairs with strong negative correlations and putative miRNA binding sites. Here, we identified a total of 37,950 potential miRNA–mRNA target pairs that involved 714 DE miRNAs. It should be noted that, of course, not all genes were potentially regulated by miRNAs. Among these immune-related DE genes, none of the 460 genes corresponding to 564 mRNAs have yet been identified as regulated by miRNAs.

The testes' immune privilege involves almost every aspect of immunological regulation, including BTB with an immunoprotective role conferred by SCs, local immunosuppression, antigen-specific immune response, and immune tolerance (6). As one of the tightest tissue barriers existing in the body of mammals, BTB is mainly formed by the junctional complex at adjacent SCs, SCs-GCs, or SCs-ECM, such as tight junctions, gap junctions, and apical ectoplasmic specializations (apical ES, a testis-specific type of adhesion junction) (27, 28). Based on KEGG pathway analysis, in the present study, we found that 39, 36, and 37 DE genes were significantly enriched in tight junctions, gap junctions, and adhesion junctions, respectively. Gow et al. (29) reported that the junctional protein CLDN11 deletion leads to a lack of tight junction strands between SCs. As analyzed by mRNA-seq and qPCR, our results showed that CLDN11 expression was increased in T1Y and was potentially regulated by five known miRNAs (miR-311-3p, miR-424-5p, miR-486-5p, miR-541-3p, and miR-6525-5p).

Furthermore, we found 11 integrin molecules (i.e., ITGA2-A6, A9, B1, B2, B7, AL, and AX), the expression of which was reduced in T1Y. Cell adhesion mediated by integrins (e.g., ITGA4, A6, A7, A9, B1) has a critical role in self-renewal and differentiation of stem cells (including spermatogonial stem cells, SSCs) within the niche through direct interaction with the molecules expressed in ECM or the ECM-rich basal membranes (30). This finding would suggest that these integrins, and particularly their significant expression in T3M, may function in maintaining an optimal microenvironment for the early spermatogenesis, especially self-renewal and differentiation of SSCs, in pre-pubertal sheep testis. Of these integrins, ITGA6 and ITGB1 have been identified as the main components of hemidesmosome, which is a special junction between SCs and ECM interfaces in the basement membrane and participates in coordinating the cellular events of spermiation and BTB restructuring during spermatogenesis via the apical ES–BTB–hemidesmosome functional axis (26). We, thus, speculate that a significant decrease in expression of ITGA6 and ITGB1, as well as their homologous genes in postpubertal sheep testes, may be mainly attributed to the disassembly of this junction and subsequent BTB restructuring, with the release of mature sperm into the lumen. In addition, the integrated analysis of miRNA–mRNA showed that the expression of ITGA6 and ITGB1 was potentially regulated by 52 and 40 known miRNAs, respectively, with elevated abundance in the T1Y group. The subsequent dual-luciferase reporter assay partially confirmed that both miR-29b and miR-1185-3p targeted ITGB1. Collectively, the results illustrate that these junction-related genes may facilitate the restructuring events involving extensive cell junctions in the seminiferous epithelium, as well as contributing to the immunological barrier function of the BTB. PIN1, one of the three known prolyl-isomerase types, was reported to play a key role in adult mice spermatogenesis. The genetic deletion of PIN1 may lead to testicular atrophy and a consequent reduction in fertility (31). Recent studies showed that PIN1 participates in the maintenance of BTB function and integrity by regulating the expression of junction proteins between SCs: connexin43 (Cx43; gap junction) and N-cadherin (CDH2; adhesion junction), and its deficiency results in the destruction of BTB integrity (32, 33). In the present study, PIN1 was identified as having a significantly increased abundance in postpubertal testes, a finding suggestive of an active role for the PIN1 gene in BTB function during sheep spermatogenesis. Nevertheless, the specific mechanism requires further clarification via experiments.

In addition to BTB, active immunosuppression is another important feature of testicular immune privilege. TGFB1, a known immunosuppressive cytokine secreted mainly by SCs, is reportedly involved in maintaining testicular immune privilege by directly or indirectly suppressing immune cell activation (3, 34). In this study, a significantly increased TGFB1 was discovered in T1Y by RNA-seq and qPCR, suggesting a potential function of TGFB1 in immunosuppressive properties in postnatal sheep testes, notably postpubertal testes, as confirmed by previous studies. Additionally, we found that its three receptors (TGFBR1-3) showed significantly decreased expression in T1Y. For TGFBR2, Western blot also confirmed that its protein abundance was significantly reduced. The subsequent immunofluorescence analysis showed high levels of TGFBR2 protein in interstitial cells in T3M and T1Y, as well as moderate expression in gonocytes and spermatogonia of T3M, and spermatogonia, spermatocytes, and spermatids of T1Y. Similarly, TGFBR2 has been expressed in spermatogonia, spermatocytes, and spermatids in human testis (35). In murine testes, TGFBR2 has been reported to exist in gonocytes and Leydig cells during fetal development (36). A previous study demonstrated that TGFBR2 expression in epididymal DCs is crucial for immunotolerance to sperm in mice epididymis, the absence of which exhibits an immune response against sperm resulting in severe epididymal leukocytosis (37). Considering these previous findings, we speculate that TGFBR2 in sheep testes may be implicated in maintaining testicular immune homeostasis, along with being involved in the regulation of the development of GCs. Moreover, our results showed that TGFB1 and its two receptors TGFBR2 and TGFBR3, are predicted to be targeted by 10, 23, and 113 putative DE miRNAs, respectively, in which TGFBR2 and TGFBR3 are coregulated by miR-15-5p and miR-7050-5p.

As macrophages are the largest immune cell population in testes, their importance for testicular immune privilege is reflected by their tolerance to the autoantigenic germ cells and the local innate immune responses against microbial infections (38). In our study, we identified CD68, a macrophage marker, expressed in Tibetan sheep testes and significantly decreased in T1Y. This indicates that macrophages also exist in sheep testes and shows a possible decrease in cell number in postpubertal testes. Accumulated evidence suggests that testicular macrophages can secrete high anti-inflammatory cytokines and express low levels of TLR signaling pathway genes, along with simultaneous inhibition of pro-inflammatory signaling pathways, such as the NF-κB pathway (39). Similarly, our results showed that 48 (e.g., TRAF6, IRAK1, IRF3, TLR4, and TLR7) and 47 DE genes (e.g., IKBKBC, CCL19, CCL21, and NFKB1) were significantly enriched in TLR and NF-κB signaling pathway, respectively. Among these genes, the vast majority were expressed at low levels (FPKM < 10) in testes at two age groups and exhibited a reduced expression in the T1Y group. By miRNA–mRNA integrated analysis, miR-146-5p was potentially targeted to six genes involved in the TLR or NF-κB pathway (TLR7, LTBR, MAP2K4, PRKCB, RIPK1, and Zap70). Fifty-one target genes potentially regulated by miR-146-5p participated in biological processes or pathways with immune responses as well, such as adaptive immune responses, leukocyte transendothelial migration, antigen processing and presentation, and the TCR signaling pathway. The miR-146 family has been widely reported to have a role in maintaining immune tolerance, including pregnancy tolerance and sperm-borne immunomodulation (16). Also, miR-146 is involved in regulating the functional tolerance of immune cell subtypes, including T cells, DCs, and macrophages (16). For example, miR-146 inhibits the maturation of DCs by reducing the expression of its surface markers. In normal testes, DCs are phenotypically immature and immunologically tolerogenic (34). Consistent with these findings, in our study, a decreased CD86 gene (a surface marker for mature DCs; FPKM < 2.5) with lower abundance was identified, suggesting that DCs exist in normal sheep testis in an inactive immature state.

**Figure 9:**
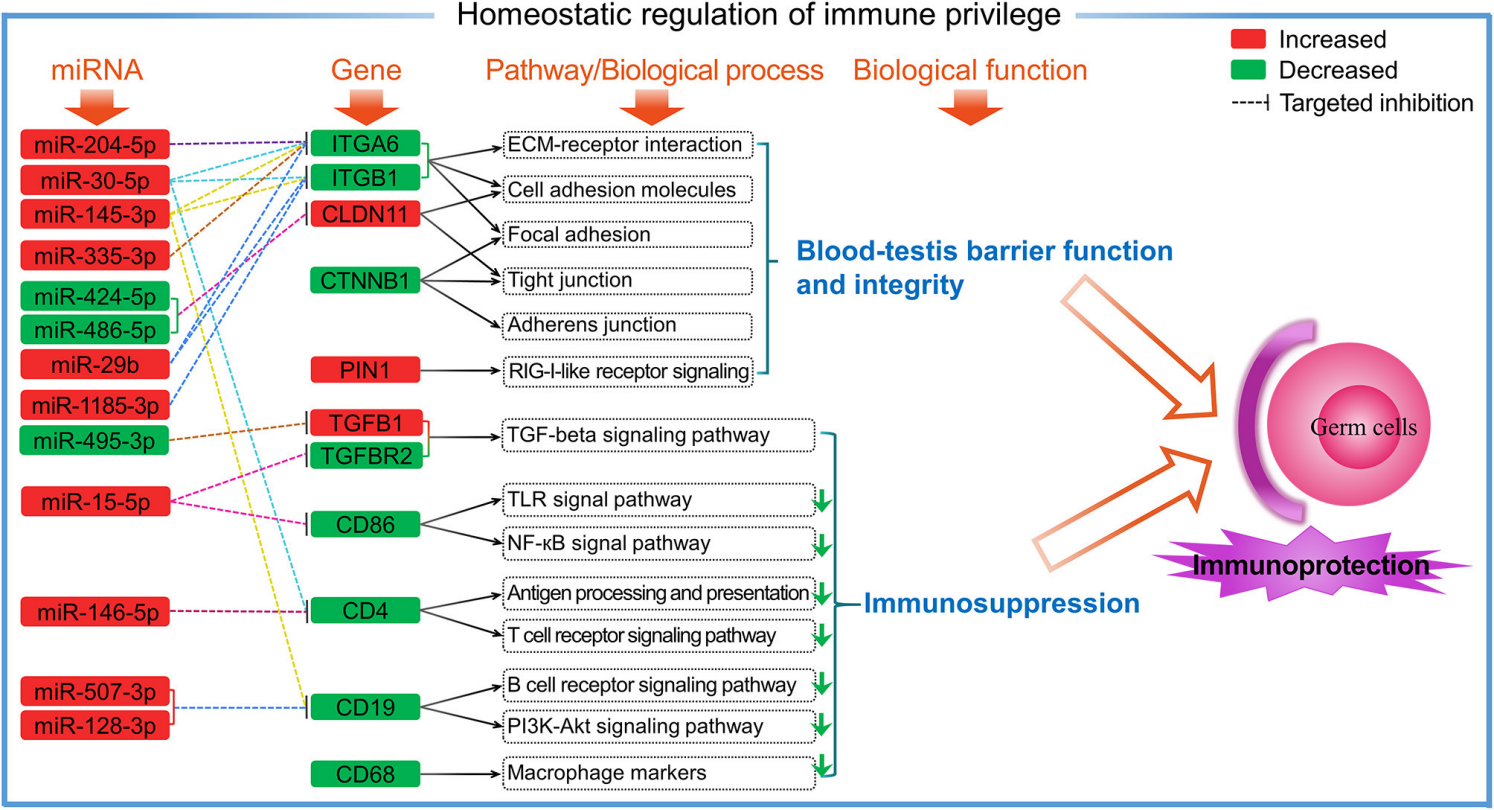
A proposed putative model showing the involvement of DE genes and miRNAs in testicular immune homeostasis through related pathways.

It has been reported that immature DCs can suppress the activation and proliferation of T cells that allow spermatogenic cells to avoid being directly recognized by the activated T cells (34). In this study, we found low CD4 expression (FPKM < 3.5 per independent sample) in both T3M and T1Y, and was decreased in T1Y. These findings suggest that the number of CD4+ T cells is kept at a very low level as well as in a quiescent state in normal sheep testes, especially in postpubertal testes, thereby preventing the potential DC-induced antigen-specific immune response. Furthermore, the present study showed that the mRNA expression and protein abundance of CD19 significantly decreased in T1Y compared with T3M. By using immunofluorescence, weak CD19 protein signals were found in interstitial cells in T3M. Interestingly, CD19 protein signals were also observed in vascular endothelium in T3M and SCs and the basement membrane in T3M and T1Y. CD19 is a known B-cell marker, but its expression is not limited to B cells, as demonstrated by the expression of CD19 in human SSCs and granulosa cells (39). Considering that CD19 is also found in the main components of BTB (SCs, basement membrane, and vascular endothelium) suggests a possible role of CD19 in the maintenance of BTB function and immune homeostasis in sheep testes. Still, the detailed mechanisms remain to be further verified. Based on the current results, a hypothetical model is proposed to reveal the potential roles of DE genes and miRNAs in the homeostatic regulation of immune privilege in sheep testes (Figure 9).

## Conclusion

To the best of our knowledge, this is the first systematic report investigating the expression patterns of miRNAs and target mRNAs related to immunity in developmental testes of sheep. Here, most of the miRNAs exhibited increased abundance, whereas most genes enriched in immune-related pathways/biological processes showed decreased abundance in postpubertal testis compared with prepubertal testis. CD19 and TGFBR2 were revealed to both exhibit reduced mRNA and protein expression. The CD19 protein was localized mainly on prepubertal testicular vascular endothelium and throughout developmental Sertoli cells and the basement membrane, whereas the TGFBR2 protein was localized primarily in interstitial cells and germ cells throughout development. Integrated analysis of miRNAs and mRNAs demonstrate that these genes, most of which may be regulated by miRNAs, participate in the homeostatic regulation of testicular immune privilege both through maintenance of the blood–testis barrier and through inhibition of the immune response and interstitial immune cell activity. Furthermore, oar-miR-29b and miR-1185-3p were confirmed to directly interact with ITGB1. These findings provide further insights into the mechanism underlying testicular immune privilege in sheep and other mammals.

## Data Availability Statement

The datasets presented in this study can be found in online repositories. The names of the repository/repositories and accession number(s) can be found in the article/Supplementary Material.

## Ethics Statement

The animal study was reviewed and approved by Laboratory Animal Welfare and Ethics Committee of Gansu Agricultural University.

## Author Contributions

TL and YM conceived and designed the study. XW, RL, and XA collected the samples. TL, XW, and RL performed the experiments and analyzed the data. TL wrote the paper. YZ, XZ, and YM contributed to revisions of the manuscript. All authors read and approved the manuscript.

## Conflict of Interest

The authors declare that the research was conducted in the absence of any commercial or financial relationships that could be construed as a potential conflict of interest.
