# Supplementary material for: Integrating miRNA and mRNA Profiling to Assess the Potential miRNA–mRNA Modules Linked With Testicular Immune Homeostasis in Sheep
Source: Front Vet Sci. 2021 May 25;8:647153. doi: 10.3389/fvets.2021.647153 (PMC8185144; doi:10.3389/fvets.2021.647153)
Supplement: Supplementary file 1 [file Table_1.DOCX]

**Table S1.** Primer information of mRNAs and miRNAs used for qPCR.

| Gene name | Primer sequence-Forward (5′-3′) | Primer sequence-Reverse (5′-3′) | Amplicon (bp) |
| --- | --- | --- | --- |
| *CD81* | ATGACCCACAGACCACCAAC | CCCAGGAACCCAACGAACAT | 127 |
| *PIN1* | GGCCGGGTGTACTACTTCAA | CCCTTGTCCATTTTTGCCGC | 93 |
| *IGF2* | CCGGACGACTTCACAGCATA | GGTGACTCTTGGCCTCTCTG | 163 |
| *CCR5* | AACCCCATCATCTACGCCTT | GTGGATCGTGTGTAAGCGG | 152 |
| *TGFBR1* | TGTCACAGATGGGCTTTGCT | AAATGGCCTGTCTCGTGGAA | 109 |
| *SMAD2* | AAGAAGTCAGCTGGTGGATC | CATCTAGAGACCTTGGTATGGT | 193 |
| *TGFBR2* | TCTTCCAAGTGACAGGCGT | TGCTCGCTGAACTCCATCA | 154 |
| *MYC* | ACACACAACGTTTTGGAGCG | AGGCGCAAGAGTTCCGTATC | 247 |
| *MAPK1* | GTCCGAGTCGCCATCAAGAA | CTGAGGTGCTGCGTCTTCAA | 221 |
| *TGFB1* | ACAATTCCTGGCGCTACCTC | ACGTCAAAGGACAGCCACTC | 73 |
| *ITGB1* | GTGTTCAGTGCCGAGCCTTCAA | CCAGCAGTCGTCAACGTCCTTC | 164 |
| *CSF1* | CCCTATGGCTGGCTTGACCT | GACATGGGCTCCAACCGAAG | 217 |
| *CSF1R* | CAGAACTGGTGCGGATTCAAGGA | GGTTGTCACGGAAGTCGGATTGT | 138 |
| *CTNNB1* | CCCTGGTGAAGATGCTTGGT | CGCACTGCCATTTTAGCTCC | 106 |
| *CD19* | TCTGCCTGACTTCCCTGGTG | TGTTTCCGTACTGGCTGTTGG | 147 |
| *IGF1* | AATCGTGGATGAGTGCTGCTT | GTTCTTGTTTCCTGCACTCCCT | 181 |
| *FAS* | CACAACTGTACTCGGACCCA | ACGTGGTGCAAGGGTTACAG | 97 |
| *FASLG* | AGCAGCCCTTGAATTACCCA | TTCAGAGGTGGTGGCGGTAG | 199 |
| *IGF1R* | ACGCCGCATCCAACAACTACATT | TTGATGGTCGTCTTCTCGCACAG | 109 |
| *CCL21* | GGACTGTTGCCTCACGTACA | TCCGAGGCGAGAACAGGATA | 116 |
| *β-actin* | CTTCCAGCCTTCCTTCCTGG | GCCAGGGCAGTGATCTCTTT | 180 |
| miR-1-3p | TTGGAATGTAAAGAAGTATGTAC | Universal reverse* | - |
| miR-30-5p | AAACATCCTACACTCTCAGCT | Universal reverse* | - |
| miR-128-3p | CACAGTGAACCGGTCTCTTT | Universal reverse* | - |
| miR-130-5p | CTCTTTCCCTGTTGCACTACT | Universal reverse* | - |
| miR-145-3p | CATTCCTGGAAATACTGTTCTT | Universal reverse* | - |
| miR-204-5p | CCCTTTGTCATCCTATGCCT | Universal reverse* | - |
| miR-340-5p | GTTATAAAGCAATGAGACTGATT | Universal reverse* | - |
| miR-497-5p | TGCAGCACACTGTGGTTTGT | Universal reverse* | - |
| miR-507-3p | TATTGGCACGTCTTGGAATGA | Universal reverse* | - |
| oar-miR-27a | TTCACAGTGGCTAAGTTCCG | Universal reverse* | - |
| miR-15-5p | TAGCAGCACATCATGGTTTAC | Universal reverse* | - |
| miR-335-3p | TTTTTCATTATTGCTCCTGACC | Universal reverse* | - |
| miR-424-5p | CCAGCAGCAATTCATGTTTTG | Universal reverse* | - |
| miR-486-5p | GTACTGAGCTGCCCCGAG | Universal reverse* | - |
| miR-146-5p | TGAGAACTGAATTCCATAGGC | Universal reverse* | - |
| oar-miR-495-3p | AAACAAACATGGTGCACTTCTT | Universal reverse* | - |
| oar-miR-29b | TAGCACCATTTGAAATCAGTGT | Universal reverse* | - |
| oar-miR-1185-3p | ATATACAGAGGGAGACTCTTAT | Universal reverse* | - |
| U6 | GGAACGATACAGAGAAGATTAGC | TGGAACGCTTCACGAATTTGCG | - |

* Universal reverse was provided by the manufacturer (Mir-X^TM^ miRNA First-Strand Synthesis Kit, Takara, Shiga, Japan).
