# Supplementary material for: Integrating miRNA and mRNA Profiling to Assess the Potential miRNA–mRNA Modules Linked With Testicular Immune Homeostasis in Sheep
Source: Front Vet Sci. 2021 May 25;8:647153. doi: 10.3389/fvets.2021.647153 (PMC8185144; doi:10.3389/fvets.2021.647153)
Supplement: Supplementary file 2 [file Table_2.DOCX]

**Table S2.** Data statistics of clean reads for each library generated by mRNA sequencing.

| Samples | Raw reads | High-quality clean reads (%) | Reads mapped to ribosomal RNAs | Unmapped reads (%) | Unique mapped reads (%) | Multiple mapped reads (%) | Mapping ratio |
| --- | --- | --- | --- | --- | --- | --- | --- |
| T3M-1 | 86686128 | 85776378 (98.95%) | 1567958 (1.83%) | 13311428 (15.81%) | 70209258 (83.38%) | 687734 (0.82%) | 84.19% |
| T3M-2 | 92873616 | 91945812 (99.00%) | 1435394 (1.56%) | 13637894 (15.07%) | 76056408 (84.03%) | 816116 (0.90%) | 84.93% |
| T3M-3 | 81045796 | 80300638 (99.08%) | 1244292 (1.55%) | 11645588 (14.73%) | 66673134 (84.34%) | 737624 (0.93%) | 85.27% |
| T3M-4 | 83913264 | 83181244 (99.13%) | 1220118 (1.47%) | 12203293 (14.89%) | 68988677 (84.17%) | 769156 (0.94%) | 85.11% |
| T1Y-1 | 80164904 | 78846662 (98.36%) | 848252 (1.08%) | 13467233 (17.27%) | 63567229 (81.50%) | 963948 (1.24%) | 82.73% |
| T1Y-2 | 75591012 | 74153400 (98.10%) | 832854 (1.12%) | 12710782 (17.34%) | 59813332 (81.58%) | 796432 (1.09%) | 82.66% |
| T1Y-3 | 79278738 | 77927550 (98.30%) | 841924 (1.08%) | 13658829 (17.72%) | 62731123 (81.38%) | 695674 (0.90%) | 82.28% |
| T1Y-4 | 83808956 | 82399922 (98.32%) | 836478 (1.02%) | 14922070 (18.30%) | 65995092 (80.91%) | 646282 (0.79%) | 81.70% |

T3M: testes from three-month-old sheep; T1Y: testes from one-year-old sheep. T3M-1, T3M-2, T3M-3, and T3M-4: biological replicates for three-month-old sheep testes; T1Y-1, T1Y-2, T1Y-3, and T1Y-4: biological replicates for one-year-old sheep testes.
