# Supplementary material for: Integrating miRNA and mRNA Profiling to Assess the Potential miRNA–mRNA Modules Linked With Testicular Immune Homeostasis in Sheep
Source: Front Vet Sci. 2021 May 25;8:647153. doi: 10.3389/fvets.2021.647153 (PMC8185144; doi:10.3389/fvets.2021.647153)
Supplement: Supplementary file 6 [file Table_6.DOCX]

**Table S6.** Data statistics of clean reads for each library generated by small RNA sequencing.

| Samples | Raw reads | High-quality clean reads (%) | Clean_tags (%) | Mapped sRNA reads | Mapping ratio |
| --- | --- | --- | --- | --- | --- |
| T3M-1 | 11169602 | 10974886 (98.26%) | 10283246 (93.70%) | 8592742 | 83.56% |
| T3M-2 | 13782333 | 13566343 (98.43%) | 13067807 (96.33%) | 10654803 | 81.53% |
| T3M-3 | 14260692 | 14002821 (98.19%) | 13373091 (95.50%) | 10856699 | 81.18% |
| T3M-4 | 15011680 | 14776485 (98.43%) | 14226473 (96.28%) | 11456489 | 80.53% |
| T1Y-1 | 14717293 | 14368303 (97.63%) | 11285556 (78.54%) | 8273553 | 73.31% |
| T1Y-2 | 14857744 | 14501959 (97.61%) | 11485427 (79.20%) | 8400681 | 73.14% |
| T1Y-3 | 13359117 | 13033906 (97.57%) | 10183585 (78.13%) | 7450791 | 73.16% |
| T1Y-4 | 13492173 | 13191822 (97.77%) | 10639443 (80.65%) | 7809735 | 73.40% |

T3M: testes from three-month-old sheep; T1Y: testes from one-year-old sheep. T3M-1, T3M-2, T3M-3, and T3M-4: biological replicates for three-month-old sheep testes; T1Y-1, T1Y-2, T1Y-3, and T1Y-4: biological replicates for one-year-old sheep testes.
